# Global practices in AT provision: considerations for a national assistive technology policy for health in India

**DOI:** 10.3389/fresc.2025.1664118

**Published:** 2025-11-10

**Authors:** Rakesh K. Srivastava, Hitesh K. Sharma, Ashoo Grover, Ruchir Malik, Ravinder Singh

**Affiliations:** 1 National Institute of Health and Family Welfare, New Delhi, India; 2 Indian Council of Medical Research, New Delhi, India

**Keywords:** assistive technology, health policy, universal health coverage, inclusive health systems, public health

## Abstract

Functional impairment refers to limitations in performing basic activities necessary for independent living, mobility, communication or social participation. Meeting the needs of people with functional impairments is an essential part of strengthening India’s health system. Present article is an attempt to cover the individuals with functional impairments under proposed Health Policy on AT, which otherwise have inadequate attention in existing public health policies, acts, and laws. Assistive technologies (AT), such as wheelchairs, white canes, hearing aids, spectacles, prosthetic limbs, communication boards, memory aids, adapted writing tools, and self-care devices, play a vital role in improving functionality, enhancing quality of life, and enabling participation in education, employment, and community activities. Based on wide range of articles reviewed from the countries with best provisioning models on AT, present health policy article proposes recommendations for a comprehensive inclusive National AT Policy for India. The policy considerations emphasize legal entitlements, sustainable financing, equitable access, integration into health systems, digital inclusion, localized manufacturing, and cross-sector collaboration. Implementing assistive technology policy is not only essential to improve public health outcomes, but also for achieving the Sustainable Development Goals by 2030 and realizing India’s vision of “Viksit Bharat” by 2047.

## 1 Introduction

As one of the world's most populous countries, the number of people with functional impairments in India is high (1). Improvements to health services and rising life expectancy mean that more people are living longer with chronic diseases, disabilities, and age-related conditions (2). The burden of functional impairments increases significantly with age, making older adults the most affected group (3). Recent nationally representative data emphasize the scale of unmet assistive technology (AT) needs in India (4). According to NFHS-5, the overall prevalence of disability is 0.93%, yet 5.11% of households include at least one person with a disability, with locomotor impairments being the most common (5, 6). Large-scale surveys highlight that the demand for AT products far exceeds current supply with over 50% of individuals with severe functional difficulties lacking access to essential AT, especially in rural and older populations (7, 8). Disparities in AT provision persist by gender, socioeconomic status, and region underscoring urgent needs for targeted interventions and enhanced data systems (9). The ongoing deployment of the rapid Assistive Technology Assessment (rATA) survey in India aims to provide robust, detailed data on unmet AT needs, regional disparities, and barriers to access, which will further inform policy and program development (4).

For these people, assistive products such as wheelchairs, hearing aids, spectacles, orthotic devices, communication boards, and mobility aids can significantly improve quality of life and support participation in education, employment, and community activities (10). Assistive products are considered a sub-set of health products as per the classification provided in the National List of Essential Assistive Products (NLEAP) developed by the Indian Council of Medical Research (ICMR) (11). In this classification, assistive products are recognized as a separate category, alongside medicines, diagnostics, medical devices, blood/cell/gene therapies, vaccines, and digital technologies, for ensuring comprehensive healthcare delivery (11).

Assistive products cover functional domains such as mobility, vision, hearing, cognition, communication, self-care, and sports/recreation/leisure activities (12, 13). They have huge potential to improve the quality of life of not only those using them, but also caretakers (14, 15). The 9As + Q + U framework (Availability, Accessibility, Affordability, Adaptability, Acceptability, Applicability, Awareness, Adherence, Assistance, Quality, and Use) and WHO Global Report on Enhancing Assistive Technology (GReAT) highlight the global need to improve access to assistive technologies (12, 16). India is actively progressing in this direction through multiple initiatives, The Rights of Persons with Disabilities Act, 2016 laid down the foundational principles for promoting the use of assistive technology for supporting persons with disabilities and ICMR is advancing this agenda through the 5Ps approach (People, Policy, Products, Personnel, and Provision) (11, 17). Policy briefs to remove barriers and enhance use of assistive technology have been developed, additionally, the ICMR Policy on Disability, Habilitation, Rehabilitation, and Assistive Care provides a comprehensive framework for facilitating accessibility and inclusion (10, 18). Several reports focusing on disability, rehabilitation and assistive technology needs have been supported by ICMR to strengthen the evidence base (19). To systematically assess the landscape, rapid data collection tools have been deployed, including the rapid Assistive Technology Assessment (rATA) for identifying unmet needs, AT-Impact for evaluating the effects of assistive products on users’ lives, and AT-Systems for measuring the readiness of service provision systems (4).

Health systems in India are not yet focused on the provision of assistive products (8). Common assistive products include wheelchairs, hearing aids, walking sticks, crutches, spectacles, prosthetic limbs, orthotic devices, and communication boards (11). Such products are made available for people with disabilities through schemes including the Scheme of Assistance to Disabled Persons for Purchase/Fitting of Aids/Appliances (ADIP) and Rashtriya Vayoshri Yojana (RVY) under the Ministry of Social Justice and Empowerment (20, 21). There are serious limitations with such an approach, as these schemes primarily target individuals who have disabilities assessed at 40% or more, as per medical certification (22). In the Indian disability framework, 40% disability signifies a benchmark level of impairment at which an individual becomes eligible for certain benefits and entitlements (23). This threshold, however, excludes many individuals with mild to moderate functional impairments who would still benefit from assistive technologies (24). As a result, the schemes do not reach all groups who need assistive products. Public health systems under different ministries including Railway Health Service (RHS) under Railways, Armed Forces Medical Services (AFMS) under Defence, Employees State Insurance (ESI) Scheme under Labour and Central Government Health Scheme (CGHS) under Health and Family Welfare will be used to strengthen AT as a health product, enabling provisioning for all those who need. The funds under various schemes will be pooled for provision of ATs. India requires a comprehensive health policy that ensures access to assistive products for everyone who needs them.

While examples from international contexts such as Australia, Canada, Norway, USA, and UK, were reviewed, the policy recommendations are rooted in India's unique demographic, socio-economic, and health system realities (25–29). These five countries were selected due to the structured availability of public data on AT systems; however, the proposed policy for India has been independently developed to suit its own needs and challenges. The selection criteria included the presence of legal frameworks supporting access to AT, public funding models, accessibility to assistive products, integration of AT into education and employment systems, financial support structures, availability of demonstration and loan programs, collaboration with private sectors, reutilization programs, digital accessibility, public awareness and advocacy initiatives, and professional training and development programs. Information on these national policies and practices was gathered through publicly available sources, including government reports, official websites, international publications, and legal documents. The information was systematically analysed across thematic areas such as legal frameworks, public funding models, accessibility to assistive products, education and employment integration, financial support and co-payment models, assistive technology demonstration and loan programs, private sector collaboration, reutilization programs, digital access and technology, public awareness and advocacy, and training and professional development. The strengths and weaknesses of each program were critically analysed to develop a robust policy proposal for India, and a summary of this analysis is provided in the supplementary information (Table 1) (25–29).

**Table 1:** Comparative overview of assistive technology provisioning systems across India and selected high-income countries.

Policy area	India	Australia	Canada	Norway	UK	USA
Legal framework	No unified national law; focus on rights through social welfare schemes	Disability Discrimination Act 1992, National Disability Strategy	Canadian Charter of Rights and Freedoms, Disability Act	Disability Rights Act, comprehensive inclusion policies	Equality Act 2010, Disability Discrimination Act (until 2010)	Americans with Disabilities Act (ADA), Assistive Technology Act (AT Act)
Public funding models	Government schemes like Ayushman Bharat, but limited AT integration	National Disability Insurance Scheme (NDIS) for AT funding	Provincial health insurance systems provide AT funding	Public insurance systems cover AT and provide subsidies for assistive devices	National Health Service (NHS) provides some AT services	Medicare, Medicaid, and private insurance reimburse AT
Accessibility to assistive products	Government and NGO programs but inconsistent access across regions	NDIS provides funding for a wide range of assistive devices	Provincial programs for AT loans and direct funding	High access to AT through public health insurance, device loans, and assistive technology centers	NHS provides direct access to devices, but waiting times exist	Loan programs, device demonstrations, and reutilization programs
Education & employment integration	Limited integration in mainstream education, focus on inclusive education needed	AT integration through government-funded programs in education and employment	Provinces provide AT in educational plans and workplace accommodations	AT provided in schools and workplaces through public funding schemes, high integration	AT integrated into schools and workplaces through public services	IDEA mandates AT in education; vocational rehabilitation programs for employment
Financial support/co-payments	Potential for subsidies or tax incentives for AT in development	Public funding for devices, some co-payments in specific programs	Publicly funded AT, some co-payments depending on province	AT funded via public insurance, minimal co-payments for most devices	Some co-payments for AT, depending on the device and region	Reimbursement models for medically necessary AT devices
AT demonstration and loan programs	Loan programs, but limited availability	Limited loan programs, but increasing availability through NDIS	Provinces have device loan programs, demonstration centers	Strong emphasis on device demonstration and loan schemes at local health centers	Limited loan programs, demonstration available via health services	iCanConnect, state-run loan and demo programs
Private sector collaboration	Growing collaboration with private companies, still in early stages	Government incentives for private sector involvement in AT	Increasing involvement of private sector in providing assistive devices	Strong public-private partnerships, especially for innovation in AT	Strong private sector involvement in device distribution	Strong private-public partnerships for device development and distribution
Reutilization programs	Limited programs for the redistribution of used devices	Reutilization encouraged through specific programs under NDIS	Some provincial programs for AT reuse	Established programs for recycling and reusing AT devices, with local health authority involvement	Limited programs, though some regional initiatives exist	National device reutilization programs, local community-based programs
Digital access and technology	Focus on assistive technology for physical disabilities, less on digital	Focus on digital inclusion under NDIS and government programs	Digital assistive technologies included in provincial funding	Strong digital access policies, with focus on making digital platforms accessible for all	Strong focus on digital access, particularly for education and employment	National initiatives for digital AT (e.g., screen readers, voice recognition)
Awareness & advocacy	Limited advocacy but growing sector	Strong advocacy, particularly in rural and underserved regions	Strong advocacy and public awareness campaigns in provinces	High public awareness programs supported by government and NGOs, strong disability advocacy	Active advocacy through non-governmental organizations (NGOs)	Strong advocacy groups, including government-funded programs for awareness
Training & professional development	Training for healthcare professionals needs expansion	Professional development programs for AT providers and support workers	Training for healthcare providers on AT provision	Ongoing training programs for healthcare providers and educators, strong collaboration with academic institutions	Specialized training programs available for educators and therapists	Focused training for healthcare providers, therapists, and educators

## 2 Considerations for national assistive technology policy for health in India

The National AT Policy for the Health Sector draws on an understanding of global practices, without positioning any specific country model as ideal, and emphasizes adapting strategies that are suitable for India's unique context (30). As per current global approaches, both the social (rights-based) model and the medical (functional impairment-based) model are used to guide the provision of rehabilitation and assistive products (31). The availability of assistive products, trained manpower, delivery through health or social systems, and models based on donor or philanthropy-driven provisioning, or scientific needs-based assessment, varies significantly across countries (32). In India, assistive products are deployed through the health system in a scattered manner. Employees in organized sectors such as defense, railways, central government, and those covered under the Employees’ State Insurance Scheme receive relatively better rehabilitation services and access to assistive products (33). However, the majority of India's population, particularly school-age children, older persons between 60 and 70 years of age, and workers in unorganized sectors such as agriculture, construction, and self-employment in urban areas, remain largely excluded from access to affordable and appropriate assistive products (33). Bridging this gap is essential for ensuring equitable health and social participation outcomes across the population.

If populations without access to assistive products are not adequately covered under suitable schemes or programs, achieving the Sustainable Development Goals (SDGs) by 2030 will not be possible (34). Assistive technologies contribute significantly toward the realization of all SDGs by promoting good health, inclusive education, economic participation, reduced inequalities, sustainable communities and strengthened partnerships, as outlined by Chapal et al. (34). Furthermore, ensuring access to assistive products is vital for advancing India's vision under the “Viksit Bharat 2047” initiative, which aims to transform India into a developed, inclusive, and prosperous nation by its 100th year of independence (35). As part of this vision, strengthening healthcare systems, ensuring universal health coverage, and promoting inclusive health policies are identified as key priorities. To ensure a comprehensive policy response, it is necessary to collect robust data on people requiring assistive products, develop a prioritized list of essential products, train skilled personnel, formulate technical standards, generate public awareness, create supportive ecosystems for manufacturing, and integrate assistive technology systematically within healthcare systems (10). Below, we outline comprehensive policy considerations for the provisioning of assistive products through India's healthcare systems.

### 2.1 Legal framework and rights-based approach

The Indian National AT Policy must ensure that access to assistive technology is recognized as a fundamental right within a robust legal framework, covering all individuals with functional impairments (30). Rather than relying solely on models from high-income countries, the policy should align with global rights-based frameworks such as the United Nations Convention on the Rights of Persons with Disabilities (UNCRPD), which India has ratified (10, 16). Nationally, the Rights of Persons with Disabilities Act, 2016, already establishes foundational principles of non-discrimination, accessibility, and equal opportunity (23). The National AT Policy should build upon this legislation to create seamless integration of assistive technology provision within healthcare services. By formally embedding AT as a legal right, India can strengthen universal health coverage, promote social inclusion, and ensure equitable access to rehabilitation and assistive products for all individuals in need (11, 36).

### 2.2 Funding and financial support models

Global experiences in public health financing were consulted to inform possible adaptations suitable for India, recognizing differences in scale, resources, and population needs **(**37). Ayushman Bharat, formally known as the Pradhan Mantri Jan Arogya Yojana (PM-JAY), is the largest health insurance scheme in the world and offers a strong foundation for integrating assistive technologies into public health coverage **(**38). While international public insurance models, such as Medicare and Medicaid in the USA, provide useful insights into financing mechanisms, it is important to recognize that India's healthcare context is characterized by a larger and more diverse population, different economic realities, and varied healthcare infrastructure. Therefore, customized adaptation is required rather than direct transplantation of such models. Challenges and limitations observed in countries like Australia, Canada and USA such as gaps in coverage and sustainability concerns, must inform the development of a tailored approach suitable for India. Financial support mechanisms could include targeted subsidies, GST exemptions, or direct support to users, inspired by elements from Australia's National Disability Insurance Scheme (NDIS) and Norway's public insurance models. Co-payment structures may also be considered to balance affordability and system sustainability. These strategies, adapted to India's socio-economic context, will help ensure that assistive technologies are accessible to all individuals who need them, regardless of socio-economic status.

Current assistive technology schemes in India, including the Scheme of Assistance to Disabled Persons for Purchase/Fitting of Aids/Appliances (ADIP) and Rashtriya Vayoshri Yojana (RVY), have significant eligibility limitations **(**30, 39). ADIP requires a minimum 40% disability certification, monthly income below Rs. 30,000, and restricts repeat benefits within three years **(**20). RVY targets only senior citizens above 60 years from Below Poverty Line families with certified age-related disabilities **(**39). These strict criteria exclude many individuals with mild to moderate functional impairments who would benefit from assistive technologies, creating substantial unmet needs. The proposed National AT Policy must expand eligibility beyond these thresholds to ensure comprehensive coverage for all individuals requiring assistive products, regardless of disability percentage or socio-economic status **(**10).

### 2.3 Access and availability of assistive products

National AT Policy must ensure that assistive technologies are readily available across both urban and rural settings in India. The policy should prioritize the development of efficient and decentralized distribution channels through existing health infrastructure, including National Institutes, Composite Regional Centres (CRCs), District Disability Rehabilitation Centres (DDRCs), and District Hospitals under the Ministry of Social Justice and Empowerment, along with Primary Health Centres (PHCs), Community Health Centres (CHCs), Sub-District Hospitals (SDHs), District Hospitals (DHs), and Medical Colleges (40, 41). Additionally, efforts should be made to establish Assistive Technology Experience Zones (AEZs) and to develop loan or rental services, enabling users to trial and borrow assistive devices before committing to purchase. These initiatives should be integrated within health and rehabilitation centers to enhance outreach, especially in underserved areas. Community-based rehabilitation (CBR) programs should be strengthened to provide local-level AT services, training, and support. Non-governmental organizations (NGOs), Disabled People's Organizations (DPOs), and community-based organizations play crucial complementary roles in last-mile delivery, awareness generation, and user feedback collection (42, 43). These organizations bridge critical gaps in government service delivery, particularly in remote and marginalized communities. Structured partnerships between public health systems and civil society organizations should be established to leverage grassroots networks, enhance community trust, and ensure culturally appropriate service delivery. CBR workers and community volunteers should be trained in basic AT assessment, fitting, and maintenance to create a sustainable local support ecosystem (18). While global experiences have demonstrated the benefits of decentralized and user-centered assistive technology programs, India's strategy must be tailored to address the diversity of its population, geographical spread, and healthcare delivery system to ensure equitable access (12).

### 2.4 Integration of AT in healthcare services

The National AT Policy must emphasize the systematic integration of assistive technologies within public health and rehabilitation services, ensuring that AT forms part of a holistic health intervention strategy (44). Assistive products should also be incorporated into India's education and para-sports systems to support inclusive development from an early stage. A multidisciplinary approach, involving doctors, therapists, rehabilitation professionals, and assistive technology specialists, should be promoted across healthcare facilities (16). These teams would be responsible for assessing individual needs, prescribing appropriate assistive products, and monitoring their usage and outcomes. Global experiences have demonstrated the value of integrating assistive technology into mainstream service delivery; however, India's approach must be adapted to its demographic diversity, healthcare infrastructure, and socio-economic conditions to ensure equitable access and long-term sustainability (41, 45).

### 2.5 Awareness, advocacy, and training

Building widespread awareness about assistive technologies is a foundational requirement for generating demand, identifying unmet needs, and ensuring the effective uptake of assistive products across India (4). The National AT Policy prioritizes structured national awareness campaigns led by the Central Health Education Bureau (CHEB) in collaboration with State Health Departments. These campaigns will have clearly defined annual targets to reach diverse populations, including rural, remote, and underserved communities. Without adequate awareness, even accessible assistive products risk underutilization (46, 47). Campaign content will address common misconceptions, such as the belief that assistive devices are only for those with severe disabilities or imply dependence. Awareness strategies will be culturally tailored to reduce stigma and promote assistive technologies as tools for empowerment, independence, and improved quality of life (47). Regular capacity-building initiatives will be institutionalized for healthcare professionals, allied health workers, and frontline service providers to ensure they are skilled in assessing, prescribing, and promoting assistive technology (48). In addition, community-based education and training modules will be developed and deployed to empower users, families, and caregivers. These coordinated efforts seek to foster a supportive environment that strengthens demand for assistive technologies and enhances uptake, thereby improving health outcomes and social inclusion for persons with functional impairments across India (49).

### 2.6 Digital inclusion and technological advancements

The National AT Policy must recognize digital assistive technologies (DAT), including text-to-speech software, speech-to-text applications, voice recognition systems, wearables, smart prosthetics, and digital communication aids, as central to enhancing accessibility and independence for individuals with functional impairments (50). India must encourage the development, local manufacturing, and adoption of these technologies to address the diverse needs of its population. Globally, the integration of digital health and telehealth solutions has expanded significantly across both high- and middle-income countries, particularly since the COVID-19 pandemic (51). India can leverage its growing digital health infrastructure to integrate telehealth services within the AT provision framework, enabling remote assessments, prescriptions, and monitoring of assistive technologies, particularly for individuals living in rural or underserved regions (52). However, significant equity challenges must be addressed, including digital literacy disparities, affordability barriers for digital AT devices, and inadequate internet connectivity in rural areas (4). The policy should include specific provisions for digital literacy training programs, subsidized internet access schemes, and low-cost digital AT solutions designed for Indian users. Leveraging the Ayushman Bharat Digital Mission infrastructure, the policy should establish tele-rehabilitation platforms, remote prescription services, and AI-enabled assistive applications optimized for diverse linguistic and cultural contexts (53). Special emphasis should be placed on developing offline-capable digital AT solutions and multi-language interfaces to ensure inclusivity across India's diverse population (4). Promoting digital inclusion will be critical for overcoming geographic barriers and ensuring that innovative assistive solutions reach all who need them, irrespective of location (54).

### 2.7 Research, innovation, and collaboration

Promotion of research into the development of new, affordable, and culturally appropriate AT through national research initiatives and collaborations with universities, private companies, and international organizations should be key focus of the National Policy (45). Like USA's partnership with tech companies in the field of AT innovation and Canada's emphasis on inclusive design, India can foster a vibrant research ecosystem that prioritizes local needs (26, 28). Similarly, like Norway's strong collaborations with industry players in healthcare innovation, Public-private partnerships will be crucial for scaling innovative solutions. International collaboration (WHO, UN, UNDP, UNICEF, AT Scale, GDI Hub etc.) also help in exchanging ideas, ensuring that the latest global advancements are accessible to the Indian population (27).

### 2.8 Evaluation, monitoring, and feedback mechanisms

Dynamic monitoring, evaluation, and feedback systems must be embedded within the National AT Policy to ensure continuous improvement, responsiveness, and long-term sustainability. These systems should be capable of systematically tracking the impact of assistive technologies on users’ health outcomes, functionality, quality of life, and accessibility to services. Periodic reviews, program audits, and structured data collection should become integral components of policy implementation. Establishing a user-centered feedback mechanism within healthcare and rehabilitation services will enable individuals with functional impairments to share their experiences, highlight challenges, and suggest areas for improvement. A robust monitoring and evaluation system will not only ensure accountability but also provide critical evidence to refine strategies, address gaps, and ensure that assistive technology services remain relevant and effective for India's diverse population.

### 2.9 Sustainability and resource management

The National AT Policy should promote systems for recycling, reutilization, and sustainable procurement of assistive products (4). Individuals should be encouraged and supported to exchange, donate, or refurbish devices that are no longer in use, ensuring that functional products reach those who might otherwise be unable to afford them (55, 56). Establishing structured recycling and reutilization programs will help reduce waste, optimize resource use, and extend the life cycle of assistive devices (56). Furthermore, the policy should emphasize sustainable procurement practices to ensure that assistive products are affordable, durable, environmentally friendly, and accessible to all socio-economic groups (34). Prioritizing resource optimization will help prevent overproduction and promote equitable distribution, ensuring that the diverse needs of India's population are efficiently and responsibly met.

### 2.10 Cross-sector collaboration

The National AT Policy must emphasize cross-sector collaboration to ensure that assistive technologies are integrated into multiple dimensions of public life (18). Effective partnerships between key ministries, including Health and Family Welfare, Education, Social Justice and Empowerment, and Science & Technology, will be essential to avoid fragmented approaches and promote a cohesive national strategy. Engagement with non-governmental organizations, civil society organizations, and advocacy groups is equally important to ensure that the voices of marginalized and underserved populations are incorporated into the policy process (57). Global experiences demonstrate that strong collaboration across sectors can enhance service delivery and improve access; however, India's approach must be tailored to its specific demographic, administrative, and socio-economic contexts. Building an inclusive and coordinated framework will be critical to ensuring that assistive technology services are accessible, equitable, and sustainable across the country (16).

Effective private sector engagement will be crucial for scaling assistive technology manufacturing, distribution, and innovation (18). The policy should establish incentive structures including tax breaks, Corporate Social Responsibility credits, and expedited regulatory approvals to stimulate local manufacturing and research (57). Structured public-private partnership models must be developed for comprehensive distribution networks, maintenance services, and technical support systems. Beyond healthcare, assistive technology integration should extend into education, employment, and transportation sectors to promote broader social inclusion and accessibility (10). Private sector collaboration will help create sustainable business models while ensuring affordability and quality of assistive products across diverse socio-economic segments (57).

### 2.11 Multi-cluster manufacturing ecosystems

A National AT Policy can catalyze the creation of a multi-cluster manufacturing ecosystem in India by fostering innovation, investment, and collaboration through targeted incentives. Tax benefits, subsidies, fast-track regulatory approvals, and Corporate Social Responsibility credits can encourage small and medium enterprises to establish specialized manufacturing units. Partnerships with academic institutions, research organizations, and international companies will drive technological advancements while ensuring affordability and quality standards. Regional clusters specializing in specific assistive technology categories can create economies of scale, generate employment, and strengthen supply chain networks. This ecosystem approach, supported by private sector engagement and cross-sectoral collaboration, will not only meet domestic demand but also position India as a global assistive technology manufacturing hub, contributing to export revenues and technological leadership.

## 3 Conclusion and future course of action

Assistive technologies will play a critical role in the health sector, especially as functional impairments rise due to aging, chronic conditions, and other health challenges. A forward-looking National AT Policy, inspired by best practices globally, can address these gaps by fostering a comprehensive approach. Legal frameworks must ensure AT access as a right, supported by sustainable funding mechanisms like public insurance, CSR, PPP, tax rebates and subsidies. Integration of AT into healthcare, employment and education, coupled with digital inclusion, will modernize service delivery. Emphasis on innovation, cross-sector collaboration, and localized manufacturing will create employment and establish India as a global leader. Training and awareness campaigns can ensure equity, reaching underserved populations. Regular evaluation and feedback systems will ensure sustainability, while resource management, including reutilization and recycling programs, will enhance affordability.

A systematic implementation roadmap is essential for realizing this policy framework. Phase 1 (Years 1–2) should focus on establishing legal foundations, expanding eligibility criteria in existing schemes, and piloting integrated AT services in select districts. Phase 2 (Years 3–5) should emphasize scaling up manufacturing ecosystems, strengthening digital AT infrastructure, and expanding coverage through Ayushman Bharat. Phase 3 (Years 6–10) should concentrate on achieving universal coverage, establishing India as a global AT manufacturing hub, and ensuring complete integration across all sectors. Key stakeholder responsibilities include: Ministry of Health and Family Welfare leading policy coordination and healthcare integration; Ministry of Social Justice and Empowerment expanding existing schemes and ensuring rights-based implementation; Ministry of Science and Technology driving innovation and manufacturing initiatives; State governments ensuring local implementation and community engagement; private sector partners contributing to manufacturing, innovation, and service delivery; and civil society organizations facilitating community mobilization and user advocacy. Success metrics should include coverage rates, quality indicators, user satisfaction measures, and impact on SDG achievements. This policy can seamlessly integrate AT into health systems, supporting India's commitment to achieving Sustainable Development Goals by 2030 and its vision for “Viksit Bharat” by 2047.
