# Supplementary material for: Global practices in AT provision: considerations for a national assistive technology policy for health in India
Source: Front Rehabil Sci. 2025 Nov 10;6:1664118. doi: 10.3389/fresc.2025.1664118 (PMC12640990; doi:10.3389/fresc.2025.1664118)
Supplement: Supplementary file 1 [file Table1.docx]

Supplementary Material

# Summary of Global Practices in Assistive Technology: Policies and Programs from Australia, Canada, Japan, Norway, UK and USA

## Australian Policies and Programs for Provisioning of Assistive Products through healthcare systems

Australia has established a robust system for the provision of assistive technology (AT) through a variety of national programs funded by government initiatives. The Commonwealth Home Support Program (CHSP) offers consumers up to $500 annually for AT, which may be increased to $1,000 at provider discretion. It focuses on self-care aids, mobility aids, communication aids, and minor home modifications to support older people in maintaining independence. The Home Care Packages (HCP) Program subsidizes care for older individuals, covering household tasks, clinical care, and AT. Similarly, the Transition Care Program (TCP) provides short-term physiotherapy, occupational therapy, and AT loans, funded through flexible care subsidies. The Short-Term Restorative Care (STRC) Program delivers up to eight weeks of tailored care, integrating AT, home modifications, and multidisciplinary support. The National Aboriginal and Torres Strait Islander Flexible Aged Care (NATSIFAC) Program focuses on personal care and equipment for rural and remote areas. Veterans benefit from the Department of Veterans’ Affairs Rehabilitation Appliances Program (RAP), which provides a wide range of AT, from mobility aids to palliative care appliances, based on eligibility under specific Acts. Additionally, the Continence Aids Payment Scheme (CAPS) assists individuals with permanent incontinence by subsidizing required products. The Australian Government Hearing Services Program funds assessments, rehabilitation, and devices for eligible individuals with hearing loss through contracted providers and manufacturers. The Stoma Appliance Scheme supplies free stoma-related products, while the Essential Medical Equipment Payment helps manage energy costs for devices like home dialysis machines and ventilators, aiding individuals with eligible medical conditions.

State and territory programs complement national initiatives, addressing local needs. For instance, the Australian Capital Territory supports AT through specialized services, while New South Wales operates programs like the Aids and Equipment Program and the Home Respiratory Program. Northern Territory features the Territory Equipment Program and SEAT services. Queensland provides funding through the Medical Aids Subsidy Scheme (MASS) for permanent disabilities, while South Australia manages equipment needs through the DHS Equipment Program. Tasmania runs the TasEquip program, and Victoria's Aids and Equipment Program offers subsidized AT, home, and vehicle modifications. Western Australia’s Community Aids and Equipment Program (CAEP) supports individuals with long-term impairments. Additionally, public insurance schemes like Medicare and the National Disability Insurance Scheme (NDIS) play a crucial role in AT provision. Medicare offers limited coverage for prostheses, continence aids, and hearing aids, mainly addressing immediate rehabilitation needs. NDIS, on the other hand, focuses on long-term support for individuals with permanent and significant disabilities, providing funding for AT purchases, rentals, and repairs. It emphasizes risk-based categorization of AT, ensuring proper assessment and usage for safety and efficacy.

Specific NDIS-funded AT includes low-risk items like non-slip mats and shower stools, as well as high-risk devices like mobility aids, powered wheelchairs, and respiratory support machines. The program also caters to children under nine with developmental delays and individuals over 65 through alternate support mechanisms. Budget allocations for NDIS highlight significant investments, including $589 million for AT capital items and $693 million for consumable or low-cost AT in 2024. Fraud detection systems, evidence advisory committees, and technological upgrades for NDIS further strengthen its operational framework. Meanwhile, aged care funding includes $531.4 million for additional home care packages to meet rising demands.

Australia’s AT provision system has several strengths, including comprehensive government-funded programs, substantial coverage through Medicare and NDIS, and dedicated state-level initiatives addressing local requirements. Advanced support systems for the aging population and integration of AT into home improvement programs further enhance its impact. However, challenges remain, such as the complex qualification process for NDIS and its focus primarily on permanent disabilities, which may exclude some beneficiaries with temporary or moderate impairments. Overall, Australia’s integrated approach to AT provision demonstrates a strong commitment to improving the quality of life for individuals requiring assistive support.

**1.1.1 Implications of Australia's policies and programs for provisioning of assistive technology (AT) in India**

India can draw valuable insights from Australia's comprehensive policies and programs for provisioning assistive products to address the growing need for assistive technology (AT) in an inclusive and sustainable manner. The following are key policy implications for India, based on Australia's approach:

1. **Establishing a Robust National Framework**

- **Australia's Example**: Programs like the National Disability Insurance Scheme (NDIS), Commonwealth Home Support Program (CHSP), and Home Care Packages (HCP) demonstrate the importance of centralized frameworks that cater to diverse needs, from short-term restorative care to long-term disability support.
- **Policy Implication for India**: India should develop a national framework for assistive technology provision under a centralized scheme (e.g., an AT Mission), integrating existing programs such as the Sugamya Bharat Abhiyan the Rights of Persons with Disabilities (RPWD) Act, 2016, various National Health Programmes, Khelo India, Digital India, and Sarv Shiksha Abhiyan (Education for All). This framework should address AT needs across healthcare, rehabilitation, and daily living.

1. **Holistic Funding Mechanisms**

- **Australia's Example**: Programs are funded through a mix of federal grants, user contributions, and subsidies. Specialized programs like the NDIS allocate substantial resources for high-cost and low-cost assistive technologies, with eligibility-based government support.
- **Policy Implication for India**: India should diversify funding sources by combining public and private investments, leveraging corporate social responsibility (CSR) funds, insurance schemes, and partnerships with multilateral agencies. Subsidies should be tailored based on socio-economic status to ensure affordability.

1. **Inclusive Eligibility Criteria**

- **Australia's Example**: Programs like CAPS and NDIS clearly define eligibility criteria based on age, disability status, and functional impairment, ensuring targeted assistance. Separate pathways exist for children (early intervention) and older adults.
- **Policy Implication for India**: Define clear eligibility criteria for AT provisioning, encompassing children with developmental delays, individuals with disabilities, and the elderly. Programs should include provisions for temporary impairments and underserved populations like rural residents, aligning with India's diverse demographic.

1. **Focused Programs for Aging and Indigenous Populations**

- **Australia's Example**: CHSP, HCP, and the National Aboriginal and Torres Strait Islander Flexible Aged Care (NATSIFAC) programs provide tailored support for older and indigenous populations, focusing on home modifications, mobility aids, and personal care.
- **Policy Implication for India**: Develop specialized programs for senior citizens under schemes like the National Program for Health Care of the Elderly (NPHCE). Additionally, include tribal and marginalized communities in AT interventions through targeted outreach programs.

1. **Leveraging Allied Health Professionals**

- **Australia's Example**: Occupational therapists (OTs), physiotherapists, and allied health teams play a crucial role in assessing, prescribing, and customizing AT.
- **Policy Implication for India**: Strengthen human resources in rehabilitation and allied health by establishing training centers under the proposed University for Disability Studies and Rehabilitation Sciences. Mandate the involvement of allied health professionals in AT assessments and prescription.

1. **Promoting Innovation in Assistive Technology**

- **Australia's Example**: Partnerships with research entities like the National Acoustic Laboratories foster innovation in assistive devices.
- **Policy Implication for India**: Create innovation hubs for AT development under organizations such as ICMR, AMTZ, and KIHT. Provide grants for startups focusing on affordable and accessible AT solutions tailored to India's population.

1. **Comprehensive AT Coverage**

- **Australia's Example**: Programs cover a wide range of AT, from mobility aids to communication devices and home modifications, with both low- and high-risk devices addressed systematically.
- **Policy Implication for India**: Expand the scope of AT covered under government programs to include modern aids like electric wheelchairs, assistive listening devices, and home automation systems. Establish a national inventory of assistive products to standardize availability and access.

1. **Public-Private Partnerships (PPP)**

- **Australia's Example**: Collaboration with private service providers, manufacturers, and insurers ensures service delivery efficiency and affordability.
- **Policy Implication for India**: Promote PPP models for AT distribution and maintenance. Collaborate with insurance providers to include AT in health insurance policies. Work with tech companies to enhance accessibility features in mainstream devices.

1. **Decentralized State-Level Implementation**

- **Australia's Example**: State-level programs such as EnableNSW and the Victorian Aids and Equipment Program address localized needs and ensure the efficient delivery of AT.
- **Policy Implication for India**: Encourage states to implement region-specific AT programs, addressing unique challenges like terrain and accessibility. This can be supported through flexible funding mechanisms and capacity-building initiatives.

1. **Simplifying Access and Reducing Bureaucracy**

- **Australia's Example**: Programs like CAPS and RAP streamline access through clear guidelines and minimal bureaucracy, ensuring timely support.
- **Policy Implication for India**: Simplify AT provisioning processes by adopting digital platforms for applications, approvals, and monitoring. Integrate AT-related services with existing health infrastructure under Ayushman Bharat.

1. **Long-Term Sustainability and Maintenance**

- **Australia's Example**: The NDIS provides funding for repairs and maintenance of AT, ensuring its long-term usability.
- **Policy Implication for India**: Establish sustainable models for AT maintenance and repair through local service centers. Include maintenance costs in government subsidies and ensure user training on AT usage.

1. **Raising Awareness and Advocacy**

- **Australia's Example**: Awareness campaigns and consultations with stakeholders promote the effective utilization of AT.
- **Policy Implication for India**: Launch nationwide campaigns to raise awareness about the benefits of AT and available programs. Engage community health workers and NGOs to spread awareness in rural and remote areas.

1. **Integration with Existing Health and Disability Policies**

- **Australia's Example**: Programs are well-integrated with Australia's universal health care system (Medicare) and disability policies.
- **Policy Implication for India**: Integrate AT provisioning with national health programs like NHM and PMJAY. Align AT strategies with India's disability rights legislation to create a unified policy framework.

1. **Monitoring and Data-Driven Policies**

- **Australia's Example**: The NDIS invests in data systems to monitor program efficiency and detect fraud.
- **Policy Implication for India**: Implement robust monitoring systems to track AT distribution, utilization, and impact. Use data analytics to identify gaps and prioritize interventions.

1. **Budget Allocation for Assistive Technology**

- **Australia's Example**: Significant budget allocations for AT in NDIS and aged care programs reflect the government’s commitment to this sector.
- **Policy Implication for India**: Increase budgetary support for AT under central and state schemes. Establish dedicated funds for research, subsidies, and scaling up AT access.

## Canadian Policies and Programs for Provisioning of Assistive Products through healthcare systems

Canada’s approach to assistive technology (AT) provision is characterized by a combination of national and provincial programs targeting specific groups, disabilities, and needs. Nationally, initiatives like the Canada Post – Literature for the Blind Program provide free postage for materials used by blind individuals, including Braille items and audio recordings. Correctional Services Canada ensures incarcerated individuals have access to mobility aids, orthoses, prosthetics, and hearing aids, accompanied by services such as assessment and repairs. The Canada Student Grant for Services and Equipment for Students with Permanent Disabilities supports students with physical or mental disabilities by funding assistive devices, offering up to CAD 20,000 per year for eligible post-secondary students. Refugees and other vulnerable groups benefit from the Interim Federal Health Program (IFHP), which provides mobility, sensory, and other assistive devices to individuals who are not Canadian citizens. Additionally, the Non-Insured Health Benefits (NIHB) Program caters to eligible First Nations and Inuit populations, covering vision care, medical supplies, mobility aids, prosthetics, and other essential assistive technologies.

The National Defense and Canadian Armed Forces Health Services and Veterans Affairs Canada (VAC) also run specialized programs. Military personnel, depending on their duty status, receive mobility and sensory aids with associated repair and replacement services. Veterans benefit from a suite of programs under VAC, such as Programs of Choice (POC) for daily living aids, hearing services, and prosthetics, ensuring comprehensive support for rehabilitation and independence.

Provincial programs further complement these national efforts. Alberta’s Aids to Daily Living Program (AADL) provides funding for medical equipment and specialized products, particularly for seniors and individuals with disabilities. Ontario’s Assistive Devices Program (ADP) subsidizes up to 75% of assistive device costs, with full coverage available for individuals receiving social assistance. Saskatchewan’s Aids to Independent Living program includes benefits for mobility devices, prosthetics, and therapeutic equipment under specific eligibility criteria. Each province tailors its programs to address regional needs, ensuring more targeted and accessible services.

Canada’s health care system, funded under the principles of the Canada Health Act, emphasizes equitable access to medically necessary services. While health care is largely provincially administered, federal funding through the Canada Health Transfer supports provincial initiatives. The system also includes tax credits and rebates for disability-related expenses, providing financial relief for individuals and families.

Canada’s strengths in AT provisioning lie in its comprehensive network of dedicated programs addressing diverse needs across different demographics and disabilities. The involvement of provincial governments ensures localized responses to health and AT requirements. However, the system faces limitations, particularly in terms of public funding for AT, leaving gaps in affordability and coverage for some individuals. Despite this, Canada’s framework exemplifies a multi-tiered approach to assistive technology provisioning, balancing national oversight with provincial flexibility to meet the unique needs of its population.

## Canadian Policies and Programs for Provisioning of Assistive Products through healthcare systems

Canada’s approach to assistive technology (AT) provision is characterized by a combination of national and provincial programs targeting specific groups, disabilities, and needs. Nationally, initiatives like the Canada Post – Literature for the Blind Program provide free postage for materials used by blind individuals, including Braille items and audio recordings. Correctional Services Canada ensures incarcerated individuals have access to mobility aids, orthoses, prosthetics, and hearing aids, accompanied by services such as assessment and repairs. The Canada Student Grant for Services and Equipment for Students with Permanent Disabilities supports students with physical or mental disabilities by funding assistive devices, offering up to CAD 20,000 per year for eligible post-secondary students. Refugees and other vulnerable groups benefit from the Interim Federal Health Program (IFHP), which provides mobility, sensory, and other assistive devices to individuals who are not Canadian citizens. Additionally, the Non-Insured Health Benefits (NIHB) Program caters to eligible First Nations and Inuit populations, covering vision care, medical supplies, mobility aids, prosthetics, and other essential assistive technologies.

The National Defense and Canadian Armed Forces Health Services and Veterans Affairs Canada (VAC) also run specialized programs. Military personnel, depending on their duty status, receive mobility and sensory aids with associated repair and replacement services. Veterans benefit from a suite of programs under VAC, such as Programs of Choice (POC) for daily living aids, hearing services, and prosthetics, ensuring comprehensive support for rehabilitation and independence.

Provincial programs further complement these national efforts. Alberta’s Aids to Daily Living Program (AADL) provides funding for medical equipment and specialized products, particularly for seniors and individuals with disabilities. Ontario’s Assistive Devices Program (ADP) subsidizes up to 75% of assistive device costs, with full coverage available for individuals receiving social assistance. Saskatchewan’s Aids to Independent Living program includes benefits for mobility devices, prosthetics, and therapeutic equipment under specific eligibility criteria. Each province tailors its programs to address regional needs, ensuring more targeted and accessible services.

Canada’s health care system, funded under the principles of the Canada Health Act, emphasizes equitable access to medically necessary services. While health care is largely provincially administered, federal funding through the Canada Health Transfer supports provincial initiatives. The system also includes tax credits and rebates for disability-related expenses, providing financial relief for individuals and families.

Canada’s strengths in AT provisioning lie in its comprehensive network of dedicated programs addressing diverse needs across different demographics and disabilities. The involvement of provincial governments ensures localized responses to health and AT requirements. However, the system faces limitations, particularly in terms of public funding for AT, leaving gaps in affordability and coverage for some individuals. Despite this, Canada’s framework exemplifies a multi-tiered approach to assistive technology provisioning, balancing national oversight with provincial flexibility to meet the unique needs of its population.

**1.2.1 Implications of Canada’s policies and programs for provisioning of assistive technology (AT) in India**

The Canadian policies and programs for assistive technology (AT) provisioning offer several valuable insights that could inform policy development in India, particularly in strengthening the accessibility, affordability, and quality of assistive products. Here are the key policy implications for India based on Canada’s approach:

- 1. **Comprehensive National and Regional Frameworks**
- **Canadian Practice**: Canada integrates national programs with provincially administered schemes to address diverse needs. National programs, such as the Non-Insured Health Benefits (NIHB) program for First Nations and Inuit, work alongside provincial initiatives like Alberta’s Aids to Daily Living (AADL) program or Ontario’s Assistive Devices Program (ADP).
- **Implication for India**: India could adopt a similar model by complementing centrally driven assistive technology initiatives with state-specific programs tailored to regional needs. A national framework like Canada’s could set uniform standards and provide central funding, while state programs could address localized challenges, ensuring inclusivity in AT provisioning.
  1. **Targeted Support for Vulnerable Groups**
- **Canadian Practice**: Specific groups, such as First Nations, Inuit, refugees, veterans, incarcerated individuals, and persons with disabilities, benefit from targeted AT policies and programs. These address mobility, sensory, and daily living needs with funding mechanisms designed for affordability.
- **Implication for India**: India's policies could incorporate targeted programs for marginalized populations, including rural communities, tribal groups, and individuals with severe disabilities. Such initiatives should ensure equitable access to AT, especially for vulnerable groups who face systemic barriers in health and rehabilitation services.
  1. **Funding Mechanisms and Cost Sharing**
- **Canadian Practice**: Programs like ADP cover 75% of the cost of assistive devices, with full coverage for individuals receiving social assistance. These systems are supplemented by external organizations (e.g., Easter Seals, Lions Clubs) for additional funding.
- **Implication for India**: India could explore cost-sharing models where government subsidies cover a substantial portion of the cost, complemented by private contributions or corporate social responsibility (CSR) initiatives. Partnerships with non-governmental organizations (NGOs) and philanthropic bodies could help bridge gaps for those unable to afford assistive products.
  1. **Centralized Insurance and Tax Incentives**
- **Canadian Practice**: Assistive products are integrated into broader health care systems funded by public insurance (e.g., Medicare) and supported by tax incentives such as disability tax credits and medical expense deductions.
- **Implication for India**: India could include assistive products under its health insurance schemes, such as Ayushman Bharat. Tax deductions and credits for assistive technology purchases and expenses could incentivize individuals and families to invest in these devices.
  1. **Focus on Community-Based Services**
- **Canadian Practice**: Programs emphasize accessibility through community-based service delivery, such as the distribution of AT through local clinics, provincial health networks, and indigenous health programs.
- **Implication for India**: A similar focus on community-based approaches would be beneficial in India. Leveraging primary health centers (PHCs), community health workers, and rehabilitation centers as distribution points for AT could improve rural and remote area access.
  1. **Integration of AT into Education and Employment Policies**
- **Canadian Practice**: Programs like the Canada Student Grant for Services and Equipment for Students with Permanent Disabilities ensure that AT supports individuals in accessing education. Additionally, assistive devices are provided to help employees with disabilities maintain productivity in the workplace.
- **Implication for India**: India could integrate AT provisioning into its education and skill development policies, ensuring that students and employees with disabilities have the tools they need. Initiatives like Skill India and NEP 2020 could include provisions for subsidizing AT to enhance learning outcomes and employment opportunities.
  1. **Recognition of Assistive Devices as Essential Health Services**
- **Canadian Practice**: AT is considered an integral part of essential health services, with its cost often covered under public health programs.
- **Implication for India**: India should recognize AT as a critical component of universal health care. This would necessitate the inclusion of AT within the scope of services provided by national health insurance programs and public health campaigns.
  1. **Diverse AT Categories and Technological Inclusion**
- **Canadian Practice**: Programs cover a broad spectrum of assistive devices, including mobility aids, prosthetics, vision aids, hearing aids, and daily living tools, while also funding repairs and replacements.
- **Implication for India**: India's AT policies should expand beyond basic devices to include a wide range of technologies that cater to diverse functional needs. Policies should also account for maintenance, repair, and replacement, ensuring long-term utility of devices.
  1. **Coordination with Non-Governmental Actors**
- **Canadian Practice**: NGOs, professional associations, and community groups collaborate with government programs to ensure delivery and funding of AT.
- **Implication for India**: India could foster partnerships with NGOs, professional associations, and CSR initiatives to create a robust network for AT distribution and training. Organizations such as Rotary International, Lions Club, and others could be key partners.
  1. **Provisions for Indigenous and Culturally Sensitive Needs**
- **Canadian Practice**: Programs like NIHB cater to the unique cultural and health needs of indigenous populations, including specific AT requirements.
- **Implication for India**: With a significant tribal population, India’s policies could incorporate culturally sensitive approaches to AT provisioning, ensuring that devices and services align with the social and cultural practices of indigenous communities.
  1. **Monitoring and Evaluation Frameworks**
- **Canadian Practice**: Programs incorporate systematic reviews and consultations to assess the effectiveness and coverage of AT provisions.
- **Implication for India**: Establishing a robust monitoring and evaluation framework to track AT distribution, utilization, and impact would help India refine its policies over time. This could involve periodic surveys, audits, and beneficiary feedback mechanisms.
  1. **Research and Development (R&D) in Assistive Technology**
- **Canadian Practice**: Programs often fund R&D efforts to advance assistive technology innovations tailored to diverse needs.
- **Implication for India**: India should prioritize R&D in assistive technology through collaborations between the government, academia, and private sector. Institutions like AMTZ (Andhra Pradesh MedTech Zone) and IITs could lead the development of cost-effective and high-quality AT solutions.
  1. **Inclusion in Rehabilitation and Long-Term Care**
- **Canadian Practice**: AT is seamlessly integrated into broader rehabilitation programs and long-term care for individuals with chronic conditions or disabilities.
- **Implication for India**: India could embed AT provisioning into its national rehabilitation framework and strengthen programs like the *Rashtriya Vayoshri Yojana* to provide AT for elderly individuals and those requiring long-term care.

**1.2.2 Conclusion**

Canada’s holistic and inclusive approach to assistive technology provisioning provides a roadmap for India to create policies that ensure equitable, affordable, and high-quality access to assistive devices. By learning from Canada’s integration of national frameworks with regional adaptability, targeted support for vulnerable populations, funding mechanisms, and community-based delivery systems, India can strengthen its own assistive technology landscape. Incorporating these strategies would align with India’s commitment to the UNCRPD (United Nations Convention on the Rights of Persons with Disabilities) and accelerate the nation’s progress toward universal health coverage and inclusivity.

## Japanese Policies and Programs for Provisioning of Assistive Products through healthcare systems

The provision of assistive technology in Japan is characterized by a well-structured system operating under multiple government-administered programs, primarily guided by the General Supports for Persons with Disabilities Act and Long-Term Care Insurance. The Assistive Device Grant System is the cornerstone of this framework, targeting individuals with disabilities who hold valid certificates issued by municipalities. This system caters to both adults and children, offering grants for a wide range of assistive devices across 18 categories, including prostheses, wheelchairs, hearing aids, and assistive communication devices. Municipalities, supported by Recovery Consultation Offices (RCOs), play a pivotal role in evaluating applications and ensuring appropriate device selection through expert consultations. RCOs comprise multidisciplinary teams, including welfare officers, doctors, physiotherapists, and prosthetists, who provide technical guidance, conduct assessments, and determine device necessity. Devices are categorized into three processes based on approval criteria, ranging from direct RCO decisions to those requiring a doctor’s recommendation or simplified municipality-based approvals. Financially, users contribute 10% of the device cost, while the remaining 90% is subsidized by the central, prefectural, and municipal governments. Income-based exemptions ensure equity, with full cost waivers for low-income households and exclusion for high-income users.

The Benefit Program for Daily Living Equipment addresses specific needs requiring device customization or adaptations. Long-Term Care Insurance, targeting users aged 65 and above or those certified as needing long-term care, facilitates in-home services through rental and purchase of equipment such as wheelchairs, walkers, special beds, and toileting aids. It also supports home modifications like handrail installations and floor leveling to accommodate assistive devices. The program covers 70-90% of costs within annual limits, encouraging accessibility for the elderly and those with chronic care needs.

The Workers’ Accident Compensation Insurance program focuses on employees affected by workplace injuries or illnesses, offering grants for assistive devices necessary for social rehabilitation and return-to-work support. Devices covered include myoelectric prostheses, Braille writing equipment, artificial larynges, and specialized beds for injury management. The expenses are subsidized through employer-paid insurance premiums, ensuring comprehensive coverage for affected workers.

Japan’s disability policies emphasize innovation, with the government committing over $650 million from 2020 to 2025 to research, development, and procurement of advanced assistive technologies. Strengths of this system include robust mechanisms to address local needs, support for workers injured on the job, and provisions for advanced devices aiding an aging population in managing daily activities. However, limitations include partial cost coverage under Long-Term Care Insurance and the burden of a 10% user contribution, which may pose financial challenges for some. Overall, Japan’s approach reflects a strong commitment to inclusivity and accessibility through government-backed frameworks, balancing technological advancements with equitable service delivery.

**1.3.1 Implications of Japan’s policies and programs for provisioning of assistive technology (AT) in India**

The Japanese model for provisioning assistive products offers valuable lessons for India, which seeks to strengthen its assistive technology (AT) ecosystem. The following policy implications can be drawn for India based on Japan’s approach:

1. **Establishing a Centralized Framework for AT Provision**

Japan’s Assistive Device Grant System and related programs operate under a unified legal framework, such as the *General Supports for Persons with Disabilities Act* and *Long-Term Care Insurance.* India could benefit from creating a similar centralized policy structure that integrates assistive technology provisioning into broader healthcare and social welfare systems. This would streamline processes, reduce duplication, and ensure uniformity in service delivery.

1. **Role of Local Governance**

Japan’s municipalities play a crucial role in approving and distributing assistive products, supported by Recovery Consultation Offices (RCOs). India can adopt a decentralized model where local government bodies, such as panchayats and municipal corporations, collaborate with disability rehabilitation centers to assess needs, approve grants, and provide assistive products. This localized approach would address regional disparities and ensure that interventions reach underserved communities.

1. **Multidisciplinary Evaluation Committees**

Japan’s RCOs employ multidisciplinary teams, including doctors, physiotherapists, prosthetists, and social workers, to assess users’ needs and recommend suitable assistive devices. India could replicate this model by establishing regional AT evaluation committees, leveraging existing health infrastructure like district hospitals and community health centers. These committees can ensure that assistive products are appropriately matched to the specific needs of users.

1. **Categorization of Assistive Devices and Simplified Approval Processes**

Japan categorizes assistive devices into those requiring detailed evaluation, medical opinions, or simplified approvals, ensuring a tiered approach to streamline service delivery. India could adopt a similar system by defining priority assistive devices that can be approved with minimal documentation while reserving detailed evaluation for complex or custom-made products. Simplified processes could include online portals for direct applications and approvals.

1. **5. Income-Based Subsidy Models**

Japan ensures equity through income-based subsidies, where low-income users are exempt from costs, and high-income users are excluded from grants. India can implement a similar sliding-scale subsidy system, linking financial assistance to income levels. This could be achieved through integration with the Aadhaar-linked Direct Benefit Transfer (DBT) system, ensuring subsidies are targeted and transparent.

1. **Integration with Insurance Systems**

Japan leverages Long-Term Care Insurance for elderly users and Workers’ Accident Compensation Insurance for occupational injuries, covering assistive products and home modifications. India could integrate assistive technology into existing insurance schemes, such as the *Ayushman Bharat - Pradhan Mantri Jan Arogya Yojana* (PM-JAY) or Employee State Insurance (ESI), to expand financial coverage for assistive devices.

1. **Promotion of Rental Models**

Japan’s rental services for short-term assistive needs, such as for progressive impairments or post-surgical rehabilitation, offer an efficient alternative to outright purchases. India can introduce rental programs for assistive products through public-private partnerships or community-based organizations, making devices accessible to users who may not need them permanently.

1. **Support for Home Modifications**

Japan’s provision for home modifications, including handrail installations and floor leveling, is a critical aspect of enabling independent living. India could launch schemes to subsidize small-scale home adaptations, especially for the elderly and persons with disabilities (PwDs), ensuring their living environments are safe and accessible.

1. **Public Investment in R&D and Innovation**

Japan’s commitment to investing over $650 million in assistive technology innovation underscores the importance of research and development. India can enhance funding for indigenous R&D through programs like the *Technology Interventions for Disabled and Elderly (TIDE)* by the Department of Science and Technology (DST), focusing on affordable and context-specific solutions.

1. **Focus on Aging Population and Geriatric Care**

With a rapidly aging population, Japan’s policies highlight the importance of integrating assistive products into geriatric care. India should anticipate similar demographic shifts by prioritizing assistive products for age-related conditions like mobility impairments, dementia, and vision loss. Programs targeting the elderly, such as the *National Program for Health Care of the Elderly (NPHCE)*, can incorporate assistive technology components.

1. **Addressing Workplace Injuries**

Japan’s Workers’ Accident Compensation Insurance provides grants for assistive devices to employees injured on the job. India can strengthen workplace injury compensation mechanisms under the Employees' Compensation Act, 1923, to include AT provisioning for rehabilitation and return to work.

1. **Multi-Tier Funding Model**

Japan’s funding model, where costs are shared between central, prefectural, and municipal governments, ensures sustainable financing for assistive technology. India can adopt a similar approach by engaging central ministries, state governments, and urban/rural local bodies in co-financing assistive product schemes.

1. **Awareness Campaigns and Capacity Building**

Japan ensures smooth and timely provisioning of assistive products through clear guidelines and public awareness. India can enhance awareness of existing schemes (e.g., *Sugamya Bharat Abhiyan* or Accessible India Campaign) and conduct capacity-building programs for government officials and healthcare workers involved in assistive technology distribution.

1. **Ensuring Equity and Inclusivity**

Japan’s inclusive policies address the needs of children, adults, and workers across various socioeconomic strata. India must ensure that assistive technology provisioning includes marginalized groups, including rural populations, women, and tribal communities, leveraging initiatives like the *Deendayal Disabled Rehabilitation Scheme (DDRS)*.

1. **Developing a Monitoring and Feedback Mechanism**

Japan’s structured monitoring, involving the Ministry of Health, Labour, and Welfare, ensures accountability in assistive technology delivery. India can develop a similar mechanism to monitor outcomes, gather user feedback, and improve service delivery. Digital tools like mobile applications can enhance user engagement and grievance redressal.

1. **Public-Private Partnerships and Social Enterprises**

Japan’s system encourages collaboration between public agencies and private manufacturers of assistive devices. India can foster similar partnerships by incentivizing startups and social enterprises to develop affordable and innovative assistive products under initiatives like *Startup India* and *Make in India*.

**1.3.2 Conclusion**

By drawing inspiration from Japan’s comprehensive approach to assistive technology provisioning, India can develop a robust and equitable system tailored to its unique socio-economic context. Policies focusing on localized implementation, income-based subsidies, insurance integration, rental models, and R&D promotion can significantly enhance access to assistive products. Additionally, addressing the needs of specific populations, such as workers, children, and the elderly, while fostering public-private collaborations, will ensure a sustainable and inclusive assistive technology ecosystem in India.

## Norwegian Policies and Programs for Provisioning of Assistive Products through healthcare systems

The provision of assistive technology (AT) in Norway is governed by the Act on Social Security, which mandates a comprehensive and structured service delivery system managed by local, regional, and national authorities. Local authorities hold the primary responsibility for health care, social services, and rehabilitation, including AT provision. At the local level, trained personnel, such as physiotherapists and occupational therapists, assess users' needs, recommend AT, and provide follow-up services. When local rehabilitation services are inadequate, users are referred to one of 13 regional assistive technology centers, which cover the entire country. These centers purchase, adapt, and deliver assistive devices to local authorities while also handling repair and maintenance. Additionally, Norway has five regional vehicle centers for vehicle-related assistive needs, and at the national level, the Norwegian Labour and Welfare Administration (NAV) operates an advisory unit to support AT service delivery.

The National Insurance Scheme (NIS) provides financial coverage for a wide range of AT, ensuring equitable access. It covers devices needed for vocational rehabilitation, education, and activation of children and young people under 26. Other provisions include guide dogs, secretarial assistance for visually impaired individuals, communication software, basic sewing patterns for clothing, and interpreter services for the deaf and deaf-blind. The service delivery process involves evaluating users' health conditions, selecting suitable AT, submitting applications to assistive technology centers, and ensuring follow-up by recommending professionals to guide users in adapting to and using the devices.

Eligibility for receiving assistive devices requires users to have a permanent functional difficulty lasting over two years, with AT meant to compensate for functional loss regardless of age. Membership in the National Insurance Scheme is mandatory, while municipalities offer short-term loans for temporary needs. NAV's disability assistance centers provide a range of services, including adaptation advice, borrowing aids for vision, hearing, reading, and writing difficulties, housing adaptations, and mobility aids. Specialized services include guide dogs, interpreting for the hearing impaired, and car allowances with adaptations. NAV also provides hearing aids, prostheses, orthoses, wigs, and sexual aids, though these require specific application processes.

Applications for AT can be submitted by individuals, professionals on behalf of residents, or experienced users with a user pass. Norway’s healthcare expenditure, at 8.1% of its GDP, reflects its commitment to health services, with substantial allocations to the National Insurance Scheme. For example, the basic amount (G) for 2024 is 124,028 kroner. Norway's AT system stands out globally for its extensive coverage, including AT for sports, with no denial of requests due to budgetary constraints.

Key strengths of Norway’s AT provisioning system include the wide range of devices covered under national health insurance and the guarantee of assistance irrespective of financial limitations. However, partial coverage requires users to bear 10% of the cost, and provisions for temporary disabilities remain limited. Despite these minor limitations, Norway’s AT system remains a model of inclusive, equitable, and efficient service delivery.

**1.4.1 Implications of Norway’s policies and programs for provisioning of assistive technology (AT) in India**

The Norwegian model for provisioning assistive technology (AT) offers several policy implications for India, particularly in fostering a more inclusive, equitable, and efficient AT service delivery system. India's efforts can be guided by Norway's structured approach, emphasizing universal access, robust financial coverage, and coordinated service delivery across different levels of governance. Below are the key policy implications for India based on Norwegian policies and programs:

1. **Universal Coverage through National Insurance**

- **Policy Implication**: Establish a national insurance scheme that provides comprehensive financial coverage for assistive products. Norway’s National Insurance Scheme (NIS) covers a wide range of AT, including those for vocational rehabilitation, education, and personal independence, irrespective of budgetary constraints. India could adopt a similar model to ensure equitable access to AT for people with disabilities (PwDs), particularly those in economically weaker sections.
- **Action Point**: Develop a central fund or expand existing schemes (e.g., Ayushman Bharat) to include AT for long-term needs like mobility aids, hearing devices, prosthetics, and assistive software.

1. **Integrated Multi-Level Governance**

- **Policy Implication**: Build a multi-tiered governance system for AT provisioning, integrating local, regional, and national levels. Norway’s system effectively decentralizes responsibilities, with local authorities conducting assessments, regional centers managing device procurement and adaptation, and national units providing advisory and technical support.
- **Action Point**: Strengthen local governance (Panchayati Raj and urban local bodies) for initial assessments and follow-ups, supported by regional AT hubs for specialized services and procurement.

1. **Comprehensive Needs Assessment and Follow-Up**

- **Policy Implication**: Train health professionals (e.g., physiotherapists, occupational therapists) to assess user needs, recommend AT, and provide follow-ups, as seen in Norway. This ensures that AT is not only provided but also effectively utilized.
- **Action Point**: Introduce training programs for healthcare workers and community rehabilitation professionals to develop expertise in AT assessment and user support, integrating it into existing health and rehabilitation services.

1. **Focus on Long-Term and Permanent Disabilities**

- **Policy Implication**: Prioritize AT provisioning for individuals with long-term or permanent functional impairments, as Norway does, while creating mechanisms for short-term loans to address temporary disabilities.
- **Action Point**: Establish clear eligibility criteria for AT provision based on disability type and duration, while enabling local authorities to manage short-term loans for temporary needs.

1. **Dedicated AT Service Centers**

- **Policy Implication**: Establish regional assistive technology centers for procurement, customization, maintenance, and repair of AT devices, modeled on Norway’s regional AT centers.
- **Action Point**: Create AT hubs at district or state levels, integrated with healthcare facilities and rehabilitation centers, to manage the end-to-end lifecycle of AT products.

1. **Financial Accessibility**

- **Policy Implication**: Ensure partial or full subsidization of AT costs for eligible individuals, addressing financial barriers. While Norway’s system requires users to bear a minor cost, such provisions can be modified to suit India’s socio-economic context.
- **Action Point**: Implement sliding-scale subsidies or complete funding for AT devices for economically disadvantaged groups, using mechanisms like Direct Benefit Transfers (DBT).

1. **Comprehensive Coverage of AT Categories**

- **Policy Implication**: Broaden the scope of AT devices covered under government schemes to include advanced devices like guide dogs, communication software, adapted vehicles, and devices for sports participation, as seen in Norway.
- **Action Point**: Update India’s existing disability-related programs (e.g., ADIP Scheme) to include innovative and modern assistive products that cater to diverse needs, including sports and recreational AT.

1. **Promotion of AT in Education and Vocational Training**

- **Policy Implication**: Emphasize AT provisioning for education, vocational training, and skill development for children and youth with disabilities, as done in Norway.
- **Action Point**: Mandate AT integration in schools, higher education institutions, and skill development programs to enhance access and participation of PwDs.

1. **User Empowerment and Simplified Processes**

- **Policy Implication**: Allow experienced AT users to directly apply for or access assistive devices, reducing bureaucracy and delays. Norway's system includes provisions for experienced users to receive user passes for direct access to AT.
- **Action Point**: Streamline application processes for AT devices in India, enabling self-service options for experienced users and online platforms for faster approvals.

1. **Sustainability and Maintenance of Devices**

- **Policy Implication**: Develop systems for the maintenance, repair, and replacement of AT devices, as Norway’s regional centers do.
- **Action Point**: Establish public-private partnerships to provide maintenance services and implement recycling programs for used or obsolete AT devices, promoting sustainability.

1. **Adequate Budget Allocation**

- **Policy Implication**: Allocate sufficient funds for AT provisioning, ensuring that requests are not denied due to budgetary constraints, as is the case in Norway.
- **Action Point**: Increase budgetary allocations for disability programs and introduce mechanisms for monitoring and efficient utilization of funds.

1. **Public Awareness and Accessibility Advocacy**

- **Policy Implication**: Conduct national campaigns to raise awareness about the availability of AT and eligibility criteria. Norway’s extensive network of NAV centers helps in disseminating information and assisting users.
- **Action Point**: Launch nationwide awareness programs involving stakeholders like NGOs, healthcare workers, and local governments to promote AT usage and services.

1. **Monitoring and Evaluation Framework**

- **Policy Implication**: Establish robust monitoring and evaluation mechanisms to assess the effectiveness of AT services, similar to Norway’s advisory unit under NAV.
- **Action Point**: Create an independent advisory body to oversee AT provisioning, conduct impact assessments, and recommend policy improvements.

1. **Encouragement of Innovation**

- **Policy Implication**: Support innovation in assistive technology through research and development, leveraging Norway’s advanced approach to device adaptation and customization.
- **Action Point**: Collaborate with academic institutions, startups, and industries to foster innovation in affordable and locally manufactured AT products.

**1.4.2 Conclusion**

Norway’s inclusive and well-structured AT provisioning system demonstrates that universal coverage, decentralized governance, professional assessments, financial accessibility, and sustained follow-up can ensure equitable access to assistive technology. By adapting these principles to India’s socio-economic and demographic context, policymakers can create a robust AT ecosystem that empowers PwDs, promotes independence, and enhances their quality of life.

**1.5** **United Kingdom: Policies and Programs for Provisioning of Assistive Products through Healthcare Systems**

**1.5.1 Summary of Policies and Programs**

The United Kingdom operates its assistive technology provisioning primarily through the National Health Service (NHS), which provides assistive products entirely free of charge, integrating them into a comprehensive and universal healthcare framework. Coverage includes a wide array of devices, such as mobility aids, prosthetics, and sensory devices. These are delivered through hospitals, clinics, and community services as part of standard NHS care. The UK allocates approximately £1.2 billion annually to assistive technology, aiming to promote equity and inclusion for individuals with disabilities.

**1.5.1.2 Specific Practices**

- Universal access to assistive products with no out-of-pocket cost for patients.
- Needs assessment, prescription, and fitting performed by qualified professionals.
- Devices distributed through hospital-based, clinic-based, and community-based channels.
- Centralized NHS coordination ensures consistency and quality across the country.
- Monitoring of impact includes reduction in hospital admissions, less dependency on long-term care, and enhanced societal inclusion and economic participation.

**1.5.1.3 Strengths**

- Free access for all eligible patients, regardless of financial status.
- Comprehensive assessment and fitting models maximize effectiveness of device provision.
- Broad range of devices and services uniformly available nationwide.

**1.5.1.4 Limitations**

- Long waiting lists are common for non-urgent assistive products, resulting in delays in service delivery for some individuals.

**1.5.2 Implications for India**

The UK’s model demonstrates the value of universal provision, central coordination, and free access to assistive products as part of standard healthcare. For India, this highlights the importance of integrating assistive technology services within the primary health system, developing financial mechanisms to support universal access, and prioritizing needs assessments and professional training for optimal service delivery. Lessons from the NHS model underscore the necessity of government commitment to funding and ongoing evaluation to promote equity, quality, and inclusion in assistive technology policy.

## United States Policies and Programs for Provisioning of Assistive Products through healthcare systems

In the United States, federal legislation has laid a strong foundation for increasing access to assistive technology (AT) for individuals with disabilities. Assistive technology was formally defined in 1987 and incorporated into the Technology-Related Assistance for Individuals with Disabilities Act (Tech Act) of 1988. Key legislations include the Americans with Disabilities Act (ADA) of 1990, which mandates accessibility in construction and employment for individuals with disabilities, and the Assistive Technology Act of 1998, amended in 2004. The latter authorizes state and territory AT programs to enhance AT provision through comprehensive initiatives, including device demonstration, loan, and reutilization programs, as well as state financing options like financial loans and reduced-cost acquisitions. The Rehabilitation Act of 1973 (amended in 1992 and 1998) and the Individuals with Disabilities Education Act (IDEA) of 2004 ensure that students with disabilities have access to a free appropriate public education (FAPE) that includes AT as part of their individualized education plans (IEPs).

Funding for AT in the U.S. spans federal, state, and private sources. Medicare, Medicaid, and private insurance often cover AT devices deemed medically necessary, such as mobility aids, diabetes supplies, and breathing equipment. Medicare, primarily serving seniors and individuals with disabilities, covers 80% of the cost of durable medical equipment (DME) prescribed for home use. Medicaid, a federal-state program for low-income individuals, varies across states but generally covers medically necessary DME and accessibility modifications like wheelchair ramps and stairlifts. Specialized programs like Early and Periodic Screening, Diagnosis, and Treatment (EPSDT) provide AT for children under Medicaid. Medicaid Waivers allow states to fund additional services, including specialized AT, under flexible provisions.

The National Deaf-Blind Equipment Distribution Program (iCanConnect), funded by the Federal Communications Commission, provides AT for individuals with significant hearing and vision loss, ensuring access to communication tools such as telephones and internet-based services. State programs like the Kentucky Assistive Technology Service (KATS) Network and Florida’s Brain and Spinal Cord Injury Program offer funding and support for AT. In New York, AT is covered under the Community First Choice Option (CFCO), with strict cost caps and detailed assessments. Minnesota's STAR program facilitates short-term and open-ended device loans and promotes AT access through Medicaid and private insurance.

Vocational Rehabilitation Services (VRS) play a vital role in supporting employment-related AT needs, funding devices that enhance job readiness and retention. Public schools under IDEA serve as primary AT funding sources for children up to age 21, ensuring AT integration into students’ IEPs. The Department of Education’s Assistive Technology Program ensures accessibility in its systems and provides AT for its employees with disabilities.

Several advocacy groups and national organizations assist individuals with disabilities in securing funding for AT, home modifications, and assessments. These include state vocational rehabilitation centers, nonprofit organizations, and federally funded programs. Collectively, these measures highlight the U.S.'s commitment to integrating AT into education, healthcare, and employment, promoting independence and inclusivity for individuals with disabilities.

**1.6.1. Implications of United States’ policies and programs for provisioning of assistive technology (AT) in India**

The U.S. policies and programs related to assistive products and technologies offer valuable insights for India as it works to enhance access to such devices. These implications span across legislative frameworks, funding models, service delivery mechanisms, and advocacy, all of which can contribute to the development of a more inclusive assistive technology ecosystem in India.

- 1. **Strengthening Legal Framework**

The U.S. has several robust federal policies such as the **Americans with Disabilities Act (ADA)** and the **Assistive Technology Act (AT Act)**, which guarantee the rights of people with disabilities and provide a structured approach to assistive technology (AT) services. India could look into formalizing similar comprehensive national legislation to improve access to AT. This legislation should focus on:

- **Non-discrimination** in access to AT across all sectors, including education, employment, healthcare, and public spaces, similar to the ADA.
- **State-level programs**: Similar to the AT Act, India could create dedicated AT programs within each state to ensure local accessibility and provision.
- **Recognition of AT as a right**: Enshrining the right to AT in national law can make provision of such devices a legally recognized service for people with disabilities.
  1. **Public Funding and Support for AT Provision**

The U.S. leverages **Medicare, Medicaid**, and private health insurance to fund AT. India could take inspiration from this model to enhance its own public funding mechanisms:

- **Expand coverage under government health schemes**: By integrating AT into existing health insurance schemes like the **Ayushman Bharat** scheme or creating a separate funding mechanism for AT devices, India can make such technologies more accessible to people with disabilities.
- **Tax benefits and subsidies**: The U.S. allows for some devices to be reimbursed or subsidized. India can consider offering similar subsidies or tax incentives for AT products, especially for low-income groups and individuals with specific disabilities.
- **Reimbursement for medical necessity**: Like in the U.S., India could introduce policies where devices are covered based on medical necessity, with clear guidelines on what qualifies for reimbursement.
  1. **Expanding Access through Loan, Demonstration, and Reutilization Programs**

The U.S. has various programs like **device loan programs, device demonstration programs**, and **device reutilization programs** that allow individuals to test, borrow, or refurbish assistive devices. India could adopt similar programs:

- **Loan programs**: India could set up community-based AT loan programs that allow individuals to borrow devices for a short period before deciding whether to purchase them.
- **Demonstration programs**: Government or non-governmental organizations could offer AT demonstration centers where people can try different devices before making a decision.
- **Reutilization programs**: India could create a system for **donating and redistributing used assistive devices** that are no longer needed, improving both sustainability and access.

1. **Integrating AT into Education and Employment Systems**

The **Individuals with Disabilities Education Act (IDEA)** in the U.S. ensures that students with disabilities receive necessary AT as part of their education. In India, similar integration of AT into educational frameworks could be beneficial:

- **Inclusive education**: Ensure that educational institutions have access to AT devices as part of the curriculum for children with disabilities. This could be done through increased funding and policy support for schools to incorporate AT into their teaching methods and tools.
- **Vocational training and employment**: As seen with U.S. vocational rehabilitation services, India could also provide **AT for employment**, especially for people with disabilities in the workforce. This includes modifying workspaces and offering AT solutions that assist in job performance.
- **Personalized plans**: Just like IDEA’s requirement of individualized education programs (IEPs), India can mandate individualized plans for people with disabilities, which include the provision of AT.

1. **Building Partnerships and Collaborations**

The **U.S. Veterans Affairs** and various **state and national programs** have established partnerships with non-profits, insurance providers, and advocacy groups to expand access to AT. India could build similar partnerships:

- **Partnerships with private sector**: India can incentivize private sector involvement in the development, distribution, and financing of assistive technologies.
- **Collaborations with NGOs**: India has a strong base of disability-focused NGOs, and collaboration with these organizations can help in creating awareness, facilitating access, and distributing AT products to underserved areas.

1. **Utilizing Technology for Accessibility**

The **National Deaf-Blind Equipment Distribution Program** (iCanConnect) in the U.S. provides equipment for communication and connectivity. India can develop similar programs to enhance digital accessibility:

- **Technology-based solutions**: India could establish initiatives that focus on **digital access** for people with disabilities, including assistive technologies for accessing the internet, social media, and communication tools.
- **Inclusive digital infrastructure**: Leveraging the country’s growing digital infrastructure to create platforms where individuals can access assistive devices, software, and services easily, with clear instructions and support.

1. **Increasing Awareness and Advocacy**

In the U.S., several advocacy groups play a key role in ensuring that individuals with disabilities receive the AT they need. India can benefit from:

- **Advocacy programs**: Empowering and supporting advocacy groups to raise awareness about the importance of AT in improving the quality of life for people with disabilities.
- **Creating a national database**: India can create a national database of available assistive technologies, similar to the U.S., to guide individuals, caregivers, and healthcare providers in selecting appropriate devices.
- **Training for professionals**: Providing training to doctors, therapists, and educators to recognize the need for AT and refer individuals to the appropriate services.

1. **Innovative Financing and Reimbursement Models**

The **Medicaid** and **Medicare** systems in the U.S. use a combination of public funding and cost-sharing mechanisms to finance AT. India could develop similar financial models:

- **Financial loan programs**: These programs can help people with disabilities access costly devices without a heavy financial burden. Partnerships with banks and microfinance institutions could support this model.
- **Co-payments**: A reimbursement system, similar to the U.S. model where people pay a portion of the cost, could be adapted in India to ensure that AT is accessible without overburdening the government.
  - 1. **Conclusion**

India can draw significant policy lessons from the U.S. in making assistive technology more accessible to its citizens with disabilities. By integrating assistive technology into education, employment, and healthcare frameworks, strengthening legislative protections, fostering public-private partnerships, and enhancing funding options, India can work toward a more inclusive society where people with disabilities are able to fully participate and thrive.
